# The Lipid Transfer Protein 1 from *Nicotiana benthamiana* Assists *Bamboo mosaic virus* Accumulation

**DOI:** 10.3390/v12121361

**Published:** 2020-11-27

**Authors:** Ling-Ying Chiu, I-Hsuan Chen, Yau-Heiu Hsu, Ching-Hsiu Tsai

**Affiliations:** 1 Graduate Institute of Biotechnology, National Chung Hsing University, Taichung 402, Taiwan; hayama1224@hotmail.com (L.-Y.C.); eva611@gmail.com (I.-H.C.); yhhsu@nchu.edu.tw (Y.-H.H.); 2 Advanced Plant Biotechnology Center, National Chung Hing University, Taichung 402, Taiwan

**Keywords:** BaMV, LTP1, chloroplast-localization, viral RNA accumulation, calmodulin-binding, dual-localization

## Abstract

Host factors play a pivotal role in regulating virus infection. Uncovering the mechanism of how host factors are involved in virus infection could pave the way to defeat viral disease. In this study, we characterized a lipid transfer protein, designated *NbLTP1* in *Nicotiana benthamiana,* which was downregulated after *Bamboo mosaic virus* (BaMV) inoculation. BaMV accumulation significantly decreased in NbLTP1-knockdown leaves and protoplasts compared with the controls. The subcellular localization of the NbLTP1-orange fluorescent protein (OFP) was mainly the extracellular matrix. However, when we removed the signal peptide (NbLTP1/ΔSP-OFP), most of the expressed protein targeted chloroplasts. Both NbLTP1-OFP and NbLTP1/ΔSP-OFP were localized in chloroplasts when we removed the cell wall. These results suggest that NbLTP1 may have a secondary targeting signal. Transient overexpression of NbLTP1 had no effect on BaMV accumulation, but that of NbLTP1/ΔSP significantly increased BaMV expression. NbLTP1 may be a positive regulator of BaMV accumulation especially when its expression is associated with chloroplasts, where BaMV replicates. The mutation was introduced to the predicted phosphorylation site to simulate the phosphorylated status, NbLTP/ΔSP/P(+), which could still assist BaMV accumulation. By contrast, a mutant lacking calmodulin-binding or simulates the phosphorylation-negative status could not support BaMV accumulation. The lipid-binding activity of LTP1 was reported to be associated with calmodulin-binding and phosphorylation, by which the C-terminus functional domain of NbLTP1 may play a critical role in BaMV accumulation.

## 1. Introduction

*Bamboo mosaic virus* (BaMV), a flexuous rod-shaped virus, has a single-stranded positive-sense RNA genome and is classified in the *Potexvirus* genus of *Alphaflexsiviridae* [1]. The genome of BaMV is 6366 nt (excluding the poly (A) tail) with a 5′-cap and 3′ poly(A) tail structure and encodes five open reading frames (ORFs) [2,3]. ORF1 encodes a 155 kDa replicase [4] containing a capping enzyme [5,6], an RNA 5′-triphosphatase, and RNA-dependent RNA polymerase (RdRp) activities [7,8]. ORFs 2 to 4 encode proteins involved in viral movement between cells [9,10]. ORF5 encodes a 25 kDa capsid protein involved in virus encapsidation, movement, and symptom development [11,12,13].

Because a virus with a small genome size accommodates a limited number of genes, the virus needs the host proteins directly or indirectly to participate in the infection cycle, including translation, replication, and spread [14,15,16]. Furthermore, the virus needs to escape from various host defense systems such as RNA silencing and hypersensitive response [17]. The expression profile of these host genes is usually tightly regulated during virus infection [18]. Therefore, screening for differentially expressed genes after virus infection could help identify the proteins required for virus infection and also those with a role against the virus. 

The cDNA amplified fragment length polymorphism (cDNA-AFLP) technique is one of the strategies used to identify differentially expressed genes after BaMV inoculation in *Nicotiana benthamiana* [19]. Rab GTPase activation protein 1 and serine/threonine kinase-like protein were found to facilitate cell-to-cell movement of BaMV [20,21]. Glutathione transferase U4 and carbonic anhydrase can assist BaMV replication [22,23]. Similarly, *Red clover necrotic mosaic virus* could hijack two small GTPases, Arf1 and Sar1, that can regulate viral RNA replication and interfere with the cellular secretory pathway [24]. Heat shock protein 70 can interact with the RdRp of *Rice stripe virus* and regulate viral replication [25].

Although lipid transfer proteins (LTPs) are widely found in plants and animals, their functions remain largely unexplored [26]. Plant LTPs are panallergens and can be divided into two main groups: nonspecific (ns) LTP1 (nsLTP1) and nsLTP2 by their molecular weight [27]. However, these two sources of LTPs have no sequence similarity [28]. Except for featuring the same eight conserved cysteines (C_1_-X_n_-C_2_-X_n_-C_3_C_4_-X_n_-C_5_XC_6_-X_n_-C_7_-X_n_-C_8_) and high isoelectric points, the two LTPs differ greatly in other properties. nsLTP1 and nsLTP2 are small (9–10 and 7 kDa, respectively) and basic, have isoelectric points of eight to nine, and are stable in environments of high temperature and wide-ranging pH [29]. Among the C_5_XC_6_ motif of the nsLTPs, the residue X is hydrophilic in nsLTP1 but hydrophobic in nsLTP2 [30,31]. nsLTP1 commonly has a large tunnel-like hydrophobic cavity at the center of its structure that is formed by four α-helices; for nsLTP2, the triangular cavity formed by helices is smaller but more flexible [32,33]. Both conformations are stabilized via four disulfide bridges composed of eight conserved cysteines [34,35].

nsLTPs can transfer various types of lipids by accommodating one of the acyl chains [36]. This property is related to many physiological functions such as cutin synthesis, pollen formation, germination, β-oxidation, and somatic embryogenesis [37,38]. nsLTPs have an antimicrobial function that can help plants defend against the invasion of fungus and bacteria [39,40]. Furthermore, nsLTPs might be involved in Ca^2+^-mediated signal transduction because they have a conserved region that can bind to calmodulin (CaM), a Ca^2+^-interacting protein. The interaction between nsLTP1 and CaM may affect the ability and efficiency of lipid transfer [41,42,43].

Most nsLTPs have a signal peptide on their N-terminus. Prediction analysis and actual observations indicate that the presence of signal peptides leads the proteins to a secretory pathway [44,45]. However, nsLTPs could also target cytosolic compartments. RlemLTP from lemon was localized on the chloroplast membrane, even though it has an extracellular signal, and the nsLTP from *Arabidopsis* was reported to localize in the cell wall [28,46].

In this study, we identified a gene, NbLTP1, that was downregulated after BaMV inoculation of *N. benthamiana* and assisted viral RNA replication. Transiently expressed NbLTP1 containing a signal peptide was secreted out of the cell as predicted. However, on removing the signal peptide, NbLTP1 associates with chloroplasts, where BaMV replicates.

## 2. Materials and Methods

### 2.1. NbLTP1 Knockdown and Virus Infection

To knock down the expression of *NbLTP1*, the *Tobacco rattle virus*-based *Agrobacterium*-mediated silencing system was used [47]. The cDNA fragment ACGT12 obtained from cDNA-AFLP in pGEM-T Easy (Promega, Madison, WI, USA) was subcloned into the pTRV2 vector with an *Eco*RI site. The resulting plasmid was designated pTRV2/NbLTP1 and transformed into *Agrobacterium* C58C1. *Agrobacterium* carrying pTRV1 or pTRV2 was cultured at 30 °C to OD_600_ = 1 and centrifuged at 3200 rpm for 20 min (Beckman, JA-25.50). The cells were resuspended in an induction buffer (10 mM MES pH 5.6, 10 mM MgCl_2_, and 150 μM acetosyringone) and incubated at 30 °C for 2 h. The two bacteria containing pTRV1 and pTRV2 were mixed in a 1:1 ratio and infiltrated by a syringe into leaves of 1-month old *N. benthamiana* plants. At approximately 10 to 14 days postinfiltration, when PDS-knockdown control plants showed a photobleach phenotype, 200 ng BaMV virion was mechanically inoculated onto the fourth leaf above the infiltrated leaves.

### 2.2. Protoplast Preparation and Viral RNA Inoculation

Approximately 2 g inoculated leaves was collected from knockdown *N. benthamiana* plants and digested with 12.5 mL filtered enzyme solution (0.1% bovine serum albumin, 0.6 mg/mL pectinase, 12 mg/mL cellulase in 0.55 M Mannitol-MES, pH 5.7) at 25 °C overnight. The mesophyll protoplasts collected from the interphase of the sucrose gradient were washed and resuspended in Mannitol-MES buffer [48]. Approximately 2.5 × 10^5^ protoplasts were inoculated with 300 ng BaMV viral RNA with 40% polyethyleneglycol-6000 and incubated at 25 °C under constant light. Total protein and RNA were extracted from these protoplasts at 24 and 48 h after incubation (see the protein and RNA extraction section).

### 2.3. Protein Extraction

Approximately 0.1 g inoculated leaf was collected and placed into a 2 mL tube containing one 6.35 mm ceramic sphere and sea sand, then the tube was frozen immediately with liquid nitrogen. The leaf tissue was ground with FastPrep-24 (MP Biomedicals, Santa Ana, CA, USA) for 20 s, vortexed with a 300 μL extraction buffer (50 mM Tris-HCl pH 8.0, 10 mM KCl, 10 mM MgCl_2_, 1 mM EDTA, 20% glycerol, 2% SDS, 10% β-mercaptoethanol) for 5 min, boiled for 5 min, vortexed for another 5 s, then centrifuged at 8500 rpm for 5 min. The soluble fraction extracted from the leaf tissue was loaded onto the gel (chlorophyll was used as the running marker). Total protein extracted from the 24 and 48 h protoplasts was similar to that used in frozen leaf tissue except mixed with a 100 μL extraction buffer and boiled for 5 min, and then 5 μL of each sample was loaded for analysis.

### 2.4. Western Blot Analysis

Total protein was extracted and separated by 12% PAGE containing 0.1% SDS. The gel was split into two parts with the upper portion containing the large RuBisCO subunit immersed in Coomassie blue solution (0.1% w/v Coomassie brilliant blue R250, 50% methanol, and 10% acetic acid) for 1 h, then destained with 10% acetic acid and 30% methanol for 1 h. The lower portion containing protein <40 kDa was transferred to a nitrocellulose membrane (PROTRAN BA 85, Schleicher and Schnell, BioScience GmbH, Dassel, Germany) and hybridized with the primary antibody against BaMV coat protein (CP), orange fluorescent protein (OFP), or actin. The membrane was washed and incubated with secondary antibody (affinity-purified goat antirabbit IgG-conjugated IRDye800; Rockland Immunochemicals, Gilbertsville, PA, USA), then fluorescence density was detected and quantified by using LI-COR Odyssey (LI-COR Biosciences, Lincoln, NE, USA).

### 2.5. Total RNA Extraction

Approximately 0.2 g leaf tissue was collected, ground to powder with liquid nitrogen, and vortexed with 1 mL TRIzol (Roche Diagnostics GmbH, Mannheim, Germany). For protoplasts, 500 μL of TRIzol was used in the RNA extraction directly. The mixture was extracted with 200 μL chloroform, kept on ice for 5 min, and centrifuged at 12,000 rpm for 15 min at 4 °C (Eppendorf 5415D). The aqueous layer containing RNAs was collected, mixed with an equal volume of isopropanol, kept on ice for 10 min, and centrifuged at 12,000 rpm for 10 min at 4 °C. The RNA pellet was washed with 70% ethanol, dried, dissolved in 10 μL deionized water, and stored at −80 °C.

### 2.6. Quantitative Real-Time RT-PCR

Approximately 1.5 μg total RNA in 1 μL water was mixed with 1.5 μL oligo dT_25_ primer (20 pmole/μL), incubated at 70 °C for 5 min, and quickly chilled on ice. To the mixture was added 7.5 μL cocktail containing a 2 μL 5× buffer (250 mM Tris-HCl pH 8.3, 375 mM KCl, and 50 mM DTT), 1.2 μL 25 mM MgCl_2_, 0.5 μL 10 mM dNTP, RNase inhibitor, and reverse transcriptase (Promega) for preincubation at 25 °C for 5 min. The reaction was incubated at 50 °C for 1 h, then transferred to 70 °C for 15 min to stop the reaction.

After enzyme activation at 95 °C for 3 min following the protocol provided by the company (KAPA SYBR FAST qPCR kit; Kapa Biosystems, Wilmington, MA, USA), PCR was carried out for 3 sec at 95 °C and 20 s at 60 °C for 40 cycles with the primer set, forward primer (5′-TAAGGCAGATGAAATATAGT-3′) and the reverse primer (5′-TAAGTAAGATCCATAATACAAC-3′) to amplify an approximately 0.2 kb fragment.

### 2.7. Transient Expression of Orange-NbLTP1 and Its Derivatives

To express NbLTP1, the coding region of NbLTP1 was amplified by PCR with total cDNA used as a template with the primer set NbLTP1/F (5′-GTCTAGAATGGCAATGGCTG-3′; *Xba*I site underlined) and NbLTP1/R (5′-GGTACCCTTGACCTTGGAG-3′; *Kpn*I site underlined) and cloned into the pGEM-T easy vector (Promega). To construct the mutant without the signal peptide, the sequence of the ORF with the removal of the first 69 nt underwent PCR with the forward primer NbLTP1/FΔSP (5′-TCTAGAATGGCTTTAACTTGTGGC-3′; *Xba*I site underlined) and reverse primer NbLTP1/R.

To construct mutants NbLTP1/ΔSP/P(-), a phosphorylation-negative mutant, the primer set was NbLTP1/P(-)F (5′-CGCCACTGACTGCTCCAAGGTC-3′) and NbLTP1/P(-)R (5′-GGGGCGATCGCGTAAGGAATATTGAC-3′); for NbLTP1/ΔSP/P(+), a phosphorylation-positive mutant, the primer set was NbLTP1/P(+)F (5′-CGACACTGACTGCTCCAAGGTC-3′) and NbLTP1/P(+)R (5′-GGGTCGATCGCGTAAGGAATATTGAC-3′); and for NbLTP1/ΔSP/CaM(-), a CaM-binding negative mutant, the primer set was NbLTP1/CaM(-)F (5′-TTGTGGTGTCAATATTCCTGCCGCGATCAGCCCCTCCACT-3′) and NbLTP1/CaM(-)R (5′-GTACTAGGGAGACCAGCTGCTGCGCCCAAATCGATTCCAGAAATAGC-3′), with the clone pGEM-T/NbLTP1/ΔSP used as a template. PCR was performed according to the protocol of the Q5 Hot Start High-Fidelity 2× Master Mix (New England Biolabs, Beverly, MA, USA). The clones were verified by sequencing and subcloned into the pEpyon vector containing OFP with *Xba*I and *Kpn*I sites. The resulting plasmids with the NbLTP1/ΔSP/P(-)-OFP, NbLTP1/ΔSP/P(+)-OFP, and NbLTP1/ΔSP/CaM(-)-OFP fusion gene were transformed into the *Agrobacterium* C58C1 strain. One day before the fusion protein was transiently expressed in *N. benthamiana* leaves, a 200 ng BaMV particle was inoculated on leaves. The accumulation of BaMV was examined at 3 dpi. *Agrobacterium* containing the fusion gene was infiltrated into *N. benthamiana* plants for 2 days, and the localization of fusion proteins was examined by confocal microscopy.

### 2.8. Confocal Microscopy

The fusion protein-expressed leaves or protoplasts were prepared and examined by confocal laser scanning microscopy (FV 1000 or FV 3000, Olympus). In brief, leaf tissues were cut into small pieces and loaded on a slide, or protoplasts were spotted on slides. The images were acquired by excitation with 543 nm HeNe Green laser and 633 nm HeNe Red laser and using different emission filter sets for detecting OFP and chloroplast auto-fluorescence.

### 2.9. Extraction of Apoplastic Wash Fluids

Approximately 0.1 g overexpressed leaf tissue was covered with cotton net and the buffer (20 mM Tris-HCl pH 8, 150 mM NaCl, 0.02% silwet L-77) to reduce the surface tension, then performed the vacuum infiltration. After being quickly dried, the leaf was wrapped with parafilm into a cylinder and transferred to 15 mL centrifuge tubes. Apoplastic wash fluid was collected after centrifuging at 1600 rpm for 15 min. The intracellular protein was extracted from the remaining leaf mentioned in protein extraction by a 300 μL extraction buffer. The protein of apoplastic wash fluid was extracted by adding a 300 μL extraction buffer then boiled for 5 min. Each sample was loaded with 5 μL for analysis.

## 3. Results

### 3.1. ACGT12 Is a cDNA Fragment of Nonspecific Lipid Transfer Protein 1 of N. benthamiana

**Figure 1:**
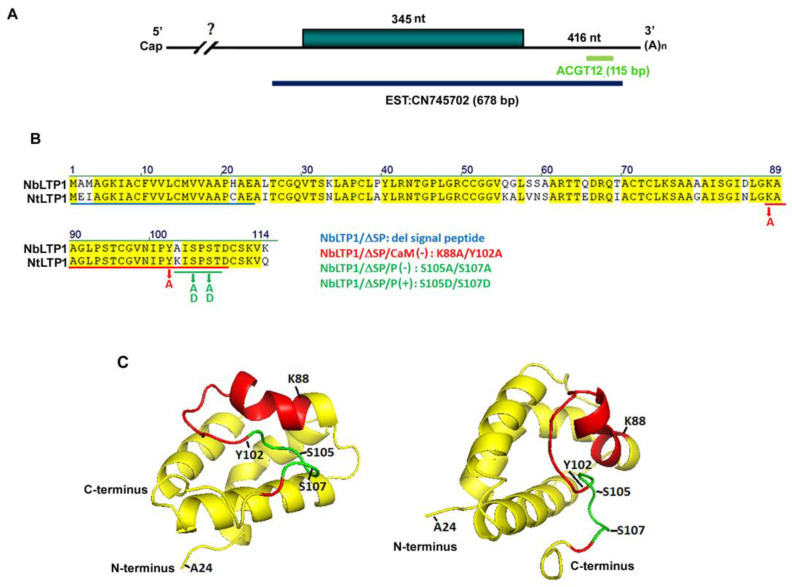
Illustration of the cDNA organization of *NbLTP1* and its structural characters. (**A**) The cDNA of *NbLTP1* with an open reading frame is indicated. The DNA fragment ACGT12 cloned from cDNA-AFLP and the DNA fragment revealed in the *N. benthamiana*-expressed sequence tag (EST with clone name CN745702) are indicated. (**B**) The sequence of NbLTP1 and alignment with NtLTP1. The alignment of the amino acid sequence of NbLTP1 derived from *Nicotiana benthamiana* (GenBank: CN745702) and *Nicotiana tabacum* (GenBank: Q42952.1). The signal peptide (1 to 23) is underlined in blue, the calmodulin-binding site is in red, and the phosphorylation signal is in green. Mutant constructs are indicated. (**C**) The three-dimensional modeling of the NbLTP1 is simulated by the Phyre2 server (http://www.sbg.bio.ic.ac.uk/~phyre2/html/page.cgi?id=index). The predicted NbLTP1 structure is visualized by PyMOL (https://pymol.org/2/).

Previously, we used the cDNA-AFLP technique to screen the differentially expressed cDNAs after BaMV infection. One of the downregulated genes containing the cDNA fragment ACGT12 (115 bp) was revealed to participate in the infection cycle of BaMV [19]. To reveal the role of this gene in BaMV infection, we cloned the full-length gene using 3′ and 5′ rapid amplification of cDNA ends (RACE). The sequence of this gene was matched to an *N. benthamiana*-expressed sequence tag (EST with clone name CN745702) containing a complete ORF (Figure 1A). The in silico translated product of the ORF showed 89 and 85% identity to nonspecific lipid transfer protein 1 (nsLTP1) of *N. sylvestris* (Accession: XP_009761744.1) and *N. tomentosiformis* (Accession: XP_009631888.1), respectively, using blastp to compare with the databases in NCBI. The sequence also showed an 85% identity with that of nonspecific lipid transfer protein 1 of *N. tabacum* (GenBank: Q42952.1) (Figure 1B) when compared with the databases in the Sol Genomics Network. We designated this gene from *N. benthamiana* as NbLTP1 in this study. The N-terminal 23 amino acids of NbLTP1 represent the signal peptide for secretion. Two sets of conserved residues (Arg/Lys at 88 and Tyr at 102 and Ser at 105 and 107) in NbLTP1 were the key residues of CaM-binding and phosphorylation sites, respectively (Figure 1B,C) [41].

### 3.2. Reduced Expression of NbLTP1 Decreases BaMV Accumulation

**Figure 2:**
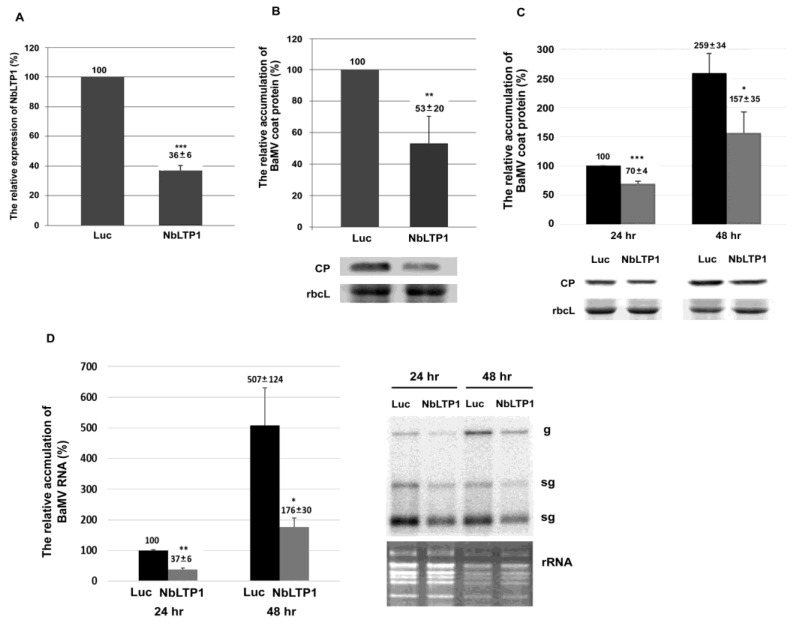
Relative accumulation of *Bamboo mosaic virus* (BaMV) coat protein (CP) in *NbLTP1*-knockdown leaves and protoplasts. (**A**) Real-time qRT-PCR of *NbLTP1* level in *NbLTP1*-knockdown and negative control (Luc) plants. (**B**) Western blot analysis of viral BaMV CP accumulation. The accumulation of BaMV CP (**C**) and RNA (**D**) was determined by Western and Northern blot analyses, respectively. The accumulation in negative control (Luc) protoplasts at 24 h postinoculation (hpi) was set to 100%. The level of rbcL was used for normalization. The accumulation in negative control plants was set to 100%. Luc: negative control; NbLTP1: *NbLTP1*-knockdown; rbcL: Rubisco large subunit (the loading control). Data are mean ± SE from at least three independent experiments. * *p* < 0.05; ** *p* < 0.01; *** *p* < 0.001 by a Student’s *t*-test.

To examine how *NbLTP1* is involved in BaMV infection, we used *Tobacco rattle virus* (TRV)-based virus-induced gene silencing to knock down the expression of *NbLTP1* in *N. benthamiana* plants. The phenotype of *NbLTP1*-knockdown plants did not differ from that of the negative control (using the *luciferase* gene fragment in the knockdown vector) plants (Figure S1). The expression of *NbLTP1* in knockdown plants was reduced to 36% of that of the control (Figure 2A), and the accumulation of BaMV CP in *NbLTP1*-knockdown leaves was significantly reduced to 53% of that of the control at 5 dpi (Figure 2B).

To distinguish whether the effect is on the cellular level or movement level, *NbLTP1*-knockdown protoplasts were inoculated with BaMV RNA. The accumulation of BaMV CP in *NbLTP1*-knockdown protoplasts was decreased to 70% and 61% of that of control protoplasts at 24 and 48 h postinoculation (hpi), respectively (Figure 2C). Northern blot analysis revealed that the accumulation of viral RNAs was reduced to 37% and 35% of that of control protoplasts at 24 and 48 hpi, respectively (Figure 2D). Thus, *NbLTP1* might be involved in BaMV accumulation.

### 3.3. NbLTP1-OFP Targets Chloroplasts in Protoplasts Even with the Removal of Its Signal Peptide

**Figure 3:**
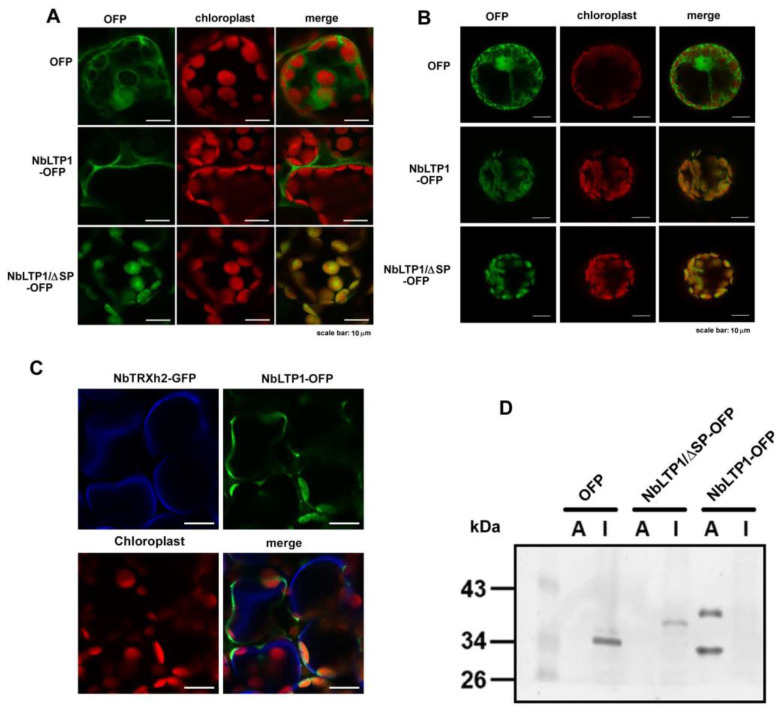
Subcellular localization and the Western blot analysis of the expression of NbLTP1-OFP and NbLTP1/ΔSP-OFP in *N. benthamiana* leaves. OFP-fused NbLTP1 and NbLTP1/ΔSP were transiently expressed in *N. benthamiana* leaves (**A**) and protoplasts (**B**). Images were obtained under an Olympus Fluoview FV1000 confocal microscope with 543 nm and 633 nm laser excitation. Scale bar is 10 μm. (**C**) High-resolution images with the plasma membrane marker (NbTRXh2-GFP) were obtained under an Olympus Fluoview FV3000 confocal microscope. Scale bar is 10 μm. (**D**) The infiltrated leaves were collected and vacuum-infiltrated with a buffer. The apoplastic fluid was collected indicated as A. The remaining tissue after removing the apoplastic fluid indicated as I for intracellular. Total protein was extracted from the apoplastic fluid (**A**) and the intracellular tissue (I).

LTPs commonly have a signal peptide at the N-terminus, which leads proteins to a secretory pathway [44,45]. Because NbLTP1 was predicted to comprise a signal peptide (Figure 1B), we assumed that NbLTP1 was localized at the extracellular space. To validate the localization of NbLTP1, we expressed the fusion protein NbLTP1-OFP in *N. benthamiana* leaves. NbLTP1 localized at the extracellular space (Figure 3A and Figure S2). The extracellular space localization was further confirmed by high-resolution images with plasma membrane marker NbTRXh2 (Figure 3C) [49]. Furthermore, NbLTP1-OFP could be isolated from the apoplastic fluid of the infiltrated leaves (Figure 3D). However, the fusion protein associated with chloroplasts when the cell wall was removed (Figure 3B). Because nsLTP from a rough lemon targets chloroplasts even though it has the secretory signal peptide, we could not rule out that NbLTP1 could localize to cytoplasmic compartments [46]. On the other hand, the construct with the signal peptide removed, NbLTP1/ΔSP-OFP, associated with chloroplasts from infiltrated leaves or protoplasts (Figure 3A,B). The high-resolution images confirmed the chloroplast localization of NbLTP1/ΔSP-OFP with the chloroplast marker PT-GK (Figure S3) [50].

### 3.4. Expression of NbLTP1/ΔSP-OFP Assists BaMV Accumulation

**Figure 4:**
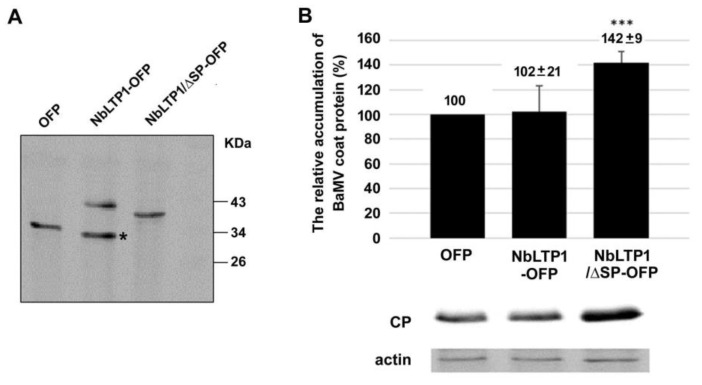
The relative accumulation of NbLTP1-OFP and NbLTP1/ΔSP-OFP in *N. benthamiana*. (**A**) Western blot analysis of the expression of NbLTP1-OFP and NbLTP1/ΔSP-OFP in *N. benthamiana* leaves. The degraded product derived from NbLTP1-OFP secreted to the extracellular space is labeled * on the blot. (**B**) The relative accumulation of BaMV CP in OFP, NbLTP1-OFP, and NbLTP1/ΔSP-OFP transiently expressed leaves. The accumulation of BaMV in OFP-expressed leaves was set to 100%. Data are mean ± SE relative levels from at least three independent experiments. *** *p* < 0.001 by a Student’s *t*-test.

The results of knockdown experiments indicated that reducing the expression of NbLTP1 decreased the accumulation of BaMV. Therefore, exogenous expression of NbLTP1 (Figure 4A) would be expected to increase BaMV accumulation. However, transient expression of NbLTP1-OFP had no effect on BaMV accumulation (Figure 4B). Because BaMV was found to replicate in chloroplasts, NbLTP1 should be transported into chloroplasts for assisting viral RNA accumulation [51]. Thus, the exogenous expression of NbLTP1 might not target chloroplasts efficiently because as shown previously, extracellular localization mainly or a limited amount of NbLTP1 is needed for the assistance. Overexpression of the chloroplast-localized NbLTP1/ΔSP-OFP (Figure 4A) significantly increased BaMV accumulation to ~142% compared with that in the control plants (Figure 4B).

The expression of NbLTP1-OFP resulted in two different polypeptides during Western blot analysis (Figure 4A). To reveal the identity of these two polypeptides, we used Edman degradation analysis to uncover their N-terminal sequence. The amino acid sequence of the 43 kDa polypeptide was LTAG (Figure S4A). The result matched that of NbLTP1 with the removal of the signal peptide, LTCG (underlined in Figure S4A). Because Edman degradation analysis did not include cysteine in the standard, Ala (control time 7.14 in Figure S4A), with the most similar retention time, was assigned to the small peak detected (indicated in Figure S4B). This result suggested that NbLTP1 was transported through the endosomal system and resulted in signal peptide removal. The polypeptide could be glycosylated through the endosomal system and led to higher molecular weight during Western blot analysis compared with NbLTP1/ΔSP (Figure 4A). The N-terminal amino acid sequence of the 34 kDa polypeptide (Figure 4A; indicated with *) is VSKG, which matched the OFP starting with the second amino acid (underlined, Figure S4B). The results suggested that this polypeptide could be the cleavage product derived from NbLTP1-OFP during exporting through the cell wall.

### 3.5. The Phosphorylation of NbLTP1 is Crucial for Efficient BaMV Accumulation

Motif analysis indicated that nsLTP1 has a signal peptide and a hydrophobic tunnel-like cavity formed by four α-helices at the N-terminal region (Figure 1C) connected with the C-terminal-conserved CaM-binding region and phosphorylation sites [32,42,43,52]. The binding of nsLTP1 to CaM as well as the phosphorylation on nsLTP1 is important for lipid binding [41,52]. To investigate whether these functional characteristics are involved in BaMV accumulation, we constructed mutants with negative phosphorylation, positive phosphorylation, and loss of CaM binding, as NbLTP1/ΔSP/P(-), -/P(+) and -/CaM(-), respectively (Figure 1B).

**Figure 5:**
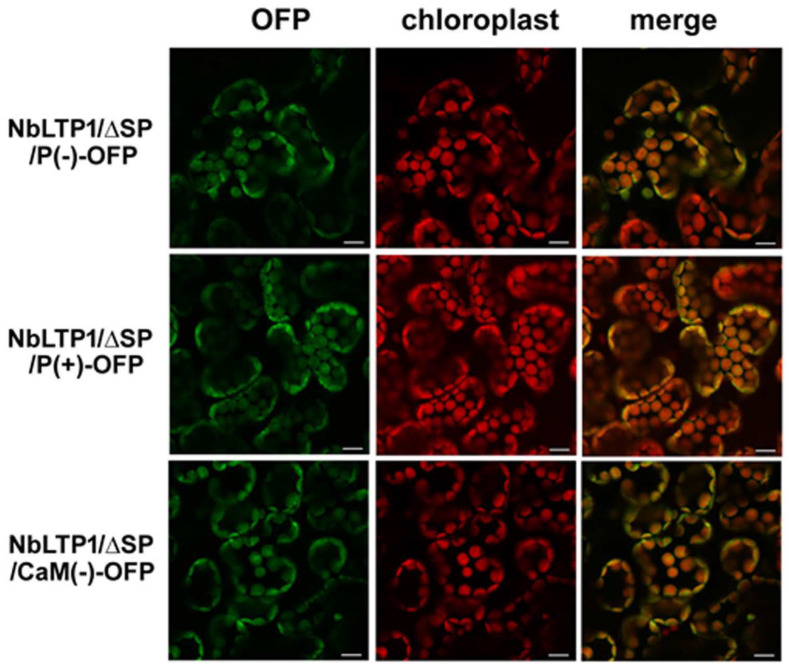
Subcellular localization of NbLTP1/ΔSP-OFP mutants in *N. benthamiana*. OFP-fused NbLTP1/ΔSP mutants were transiently expressed by agroinfiltration in *N. benthamiana* leaves. Images were obtained under an Olympus Fluoview FV1000 confocal microscope with 543 nm and 633 nm laser excitation. OFP is shown in green, and the autofluorescence of chloroplasts is in red. Scale bar is 10 μm.

**Figure 6:**
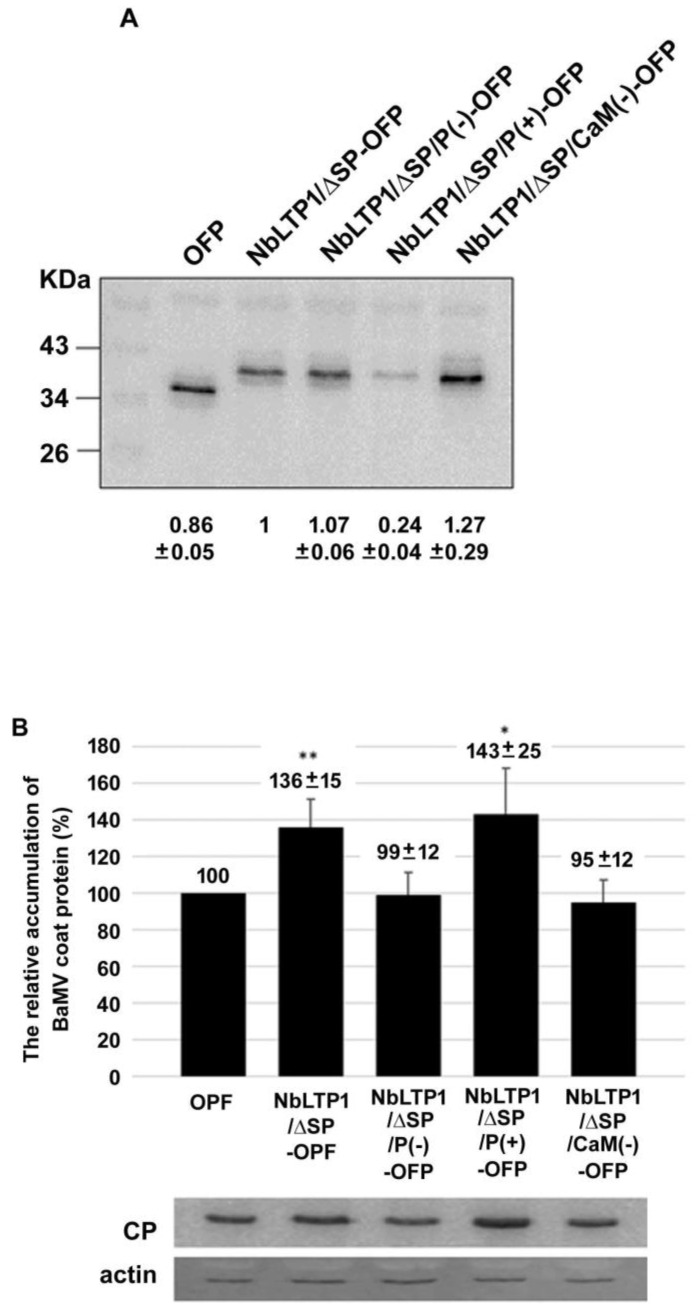
Relative accumulation of BaMV CP in NbLTP1/ΔSP-OFP and its derivatives transiently expressed in *N. benthamiana*. (**A**) Western blot analysis of protein levels of NbLTP1/ΔSP-OFP and its derivatives transiently expressed in *N. benthamiana* leaves by agroinfiltration. (**B**) The relative accumulation of BaMV CP in NbLTP1/ΔSP-OFP and its derivatives transiently expressed in leaves. The accumulation of BaMV in OFP-expressed leaves was set to 100%. Data are mean ± SE relative levels from at least three independent experiments. * *p* < 0.05, ** *p* < 0.01 by a Student’s *t*-test.

Confocal images of the localization of these constructs expressed in cells indicated that NbLTP1/ΔSP/P(-), -/P(+), and -/CaM(-) were associated with chloroplasts (Figure 5). Because of no signal peptide for secretion, they were delivered to the chloroplasts for the delivery of NbLTP1/ΔSP (Figure 4) via an unidentified mechanism. After transient expression of these constructs in *N. benthamiana* leaves (Figure 6A), BaMV was inoculated into these leaves. The accumulation of BaMV in NbLTP1/ΔSP-OFP- and -/ΔSP/P(+)-OFP-expressed leaves was approximately 136% and 143%, respectively, of that of control leaves (Figure 6B). By contrast, the BaMV accumulation in NbLTP1/ΔSP/P(-)-OFP- and -/CaM(-)-OFP-expressed leaves did not differ from that in control leaves. Thus, only the constructs competent to bind CaM and to be phosphorylated could assist in the accumulation of BaMV.

## 4. Discussion

To successfully invade its host, a viral pathogen needs to overcome the host defense mechanisms and access the host protein efficiently for assisting in viral multiplication. Revealing strategies against the viral pathogens requires identifying these host proteins with a role against or assisting the viral pathogens. In this study, we identified a downregulated gene, NbLTP1, that was demonstrated to assist in BaMV accumulation by knockdown experiments (loss of function) (Figure 2) and transient expression experiments (gain of function) (Figure 4 and Figure 6). However, the assistance occurred only when the protein NbLTP1/ΔSP-OFP was delivered to chloroplasts (Figure 3 and Figure 5), where BaMV replicates.

The major function of NbLTP1 is to transfer lipids between membranes, and the activity can be regulated by Ca^2+^-binding CaM [43]. The binding of CaM at the conserved C-terminal CaM-binding domain was demonstrated to regulate the lipid binding activity of LTPs, even though the effects differ between dicot and monocot species [41]. LTPs in dicot species, such as NbLTP1 binding CaM in the presence of Ca^2+^ via the conserved contact residue Tyr102 (Figure 1B,C), lead to cavity opening and prompt lipid binding [41]. The LTP CaM-binding protein 10, isolated from Chinese cabbage, could be phosphorylated by calcium-dependent protein kinase (CDPK), showing diminished CaM-binding ability and enhanced lipid-binding activity [52]. LTPs in plants can bind two CaM isoforms, convergent and divergent, dependent on and independent of Ca^2+^, respectively [42]. The activation of CDPK to target CaM-binding protein requires Ca^2+^. Because the phosphorylation site of NbLTP1 overlaps the CaM-binding site (Figure 1B,C), the two characteristics (CaM binding and phosphorylation) could be associated with structural regulation by Ca^2+^ signaling to activate CaM binding on NbLTP1 and result in C-terminal phosphorylation. The consequence of NbLTP1 phosphorylation is improved lipid binding, which increases BaMV accumulation (Figure 6).

LTP1 from tobacco can bind a receptor on the plasma membrane in the form of an LTP–lipid complex and then present its antifungal activity [53]. For subcellular localization, NbLTP1 was mainly secreted to the extracellular space (Figure S2); however, the localization could be reoriented to chloroplasts when the cell wall was removed or the protein lacked the signal peptide (Figure 3). Thus, NbLTP1 may process two localization signals: a major extracellular localization directed by the signal peptide and a minor chloroplast localization directed by an unknown mechanism. Some LTPs possess two subcellular localizations for direct membrane contact sites between the chloroplast outer envelope and the other cytosolic compartments for lipid transfer [54].

Most of the nuclear-encoded chloroplast proteins use the conventional way to import into chloroplast via the transit peptide recognition and pass through the chloroplast outer and inner membrane channel, translocon [55,56]. Proteomic studies of the *Arabidopsis* chloroplast indicated that approximately more than 8% of the proteins containing the predicted signal peptides for ER translocation [57]. Arabidopsis carbonic anhydrase 1 was revealed to localize in the chloroplast via an alternative route through the N-glycosylated secretory pathway [58]. The rice nucleotide pyrophosphatase/phosphodiesterase and the α-amylase isoform I-1 were the two glycoproteins targeting chloroplasts from the ER-Golgi through the secretory pathway [59,60]. The potential of NbLTP1 targeting the chloroplast could also be through the secretory pathway.

In conclusion, herein, we have identified a gene from *N. benthamiana*, *NbLTP1*, that could facilitate BaMV accumulation. The lipid-transfer activity of NbLTP1 is possibly regulated by CaM-binding and phosphorylation at its C-terminus to target the chloroplasts, from which the lipid-transfer could provide a better environment (the membrane) for viral RNA accumulation. However, the detailed mechanism still needs to be revealed.

## Supplementary Materials

The following are available online at https://www.mdpi.com/1999-4915/12/12/1361/s1, Figure S1: Morphology and silencing efficiency of *NbLTP1*-knockdown and the negative knockdown plants. The *phytoene desaturase* (PDS)-knockdown plant was a positive control, and the *luciferase* gene fragment in the knockdown vector was a negative control. Figure S2: Localization of NbLTP1-OFP in *Nicotiana benthamiana* by confocal microscopy. Proteins were transiently expressed by agroinfiltration in *N. benthamiana* leaves. Images were obtained under an Olympus Fluoview FV1000 confocal microscope with 488 nm and 633 nm laser excitations. Scale bar is 30 μm. Figure S3: Subcellular localization of NbLTP1/ΔSP-OFP in *N. benthamiana*. OFP-fused NbLTP1/ΔSP mutants were transiently expressed by agroinfiltration in *N. benthamiana* leaves. High-resolution images with the chloroplast marker (PT-GK) were obtained under an Olympus Fluoview FV3000 confocal microscope with 543 nm and 633 nm laser excitation. GFP is shown in blue, OFP is shown in green, and the autofluorescent of chloroplasts is in red. Scale bar is 10 μm. Figure S4: N-terminus sequence determination of the secreted polypeptides. Edman degradation assay was used to determine the N-terminal sequence of the two polypeptides (Figure 3C) isolated from the transiently expressed NbLTP1-OFP on *N. benthamiana* leaves. The N-terminus sequence of the larger band in (A) with the profile of the third cycle in (B) and the lower band in (C). The matched sequence of NbLTP1 in (A) and OFP in (C) is underlined. The ambiguity of the small peak in the third cycle of Edman degradation is indicated with an arrow in (B).
