# Supplementary material for: The Lipid Transfer Protein 1 from Nicotiana benthamiana Assists Bamboo mosaic virus Accumulation
Source: Viruses. 2020 Nov 27;12(12):1361. doi: 10.3390/v12121361 (PMC7760991; doi:10.3390/v12121361)
Supplement: Supplementary file 1 [file viruses-12-01361-s001.pdf]

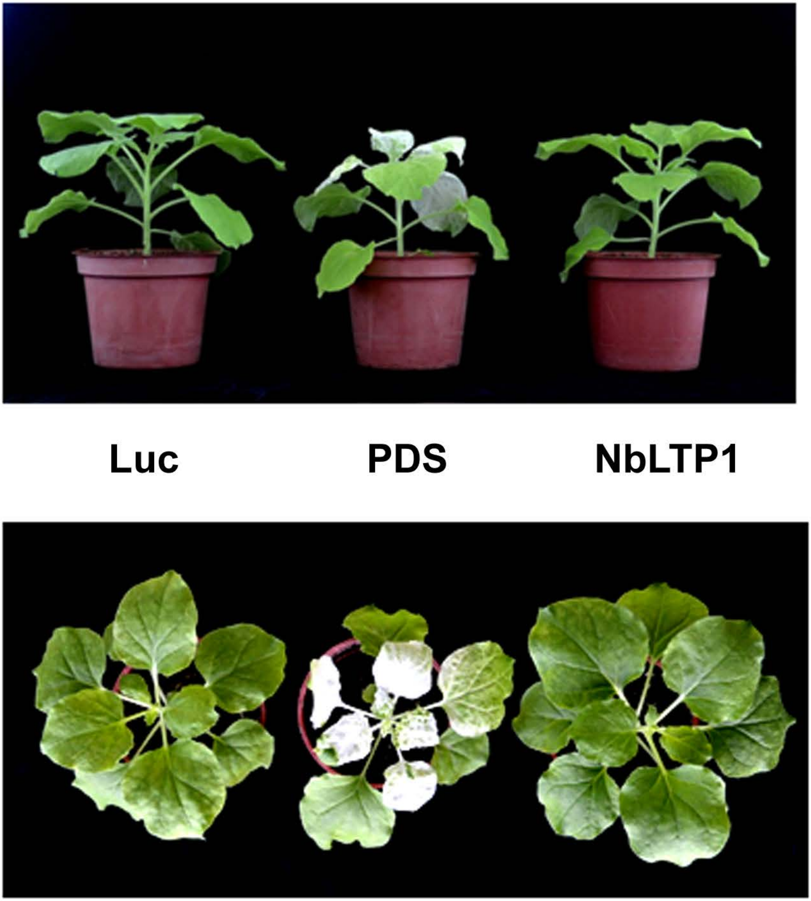

Figure S1: Morphology and silencing efficiency of *NbLTP1*-knockdown and the negative knockdown plants. The *phytoene desaturase* (PDS)-knockdown plant was a positive control and the *luciferase* gene fragment in the knockdown vector was a negative control.

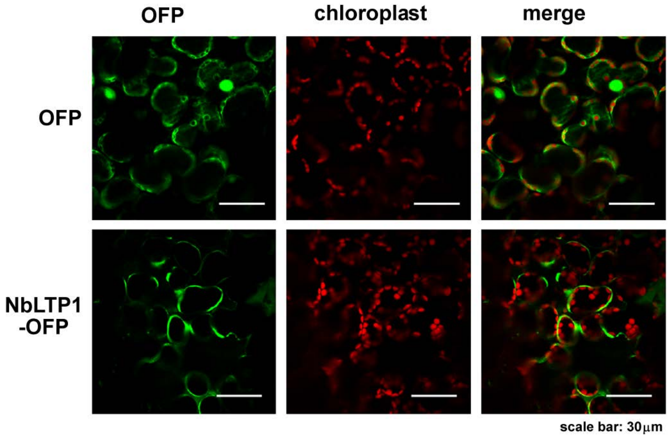

Figure S2: Localization of NbLTP1-OFP in *Nicotiana benthamiana* by confocal microscopy. Proteins were transiently expressed by agro-infiltration in *N. benthamiana* leaves. Images were obtained by under an Olympus Fluoview FV1000 confocal microscope with 488-nm and 633-nm laser excitations. Scale bar is 30 μm.

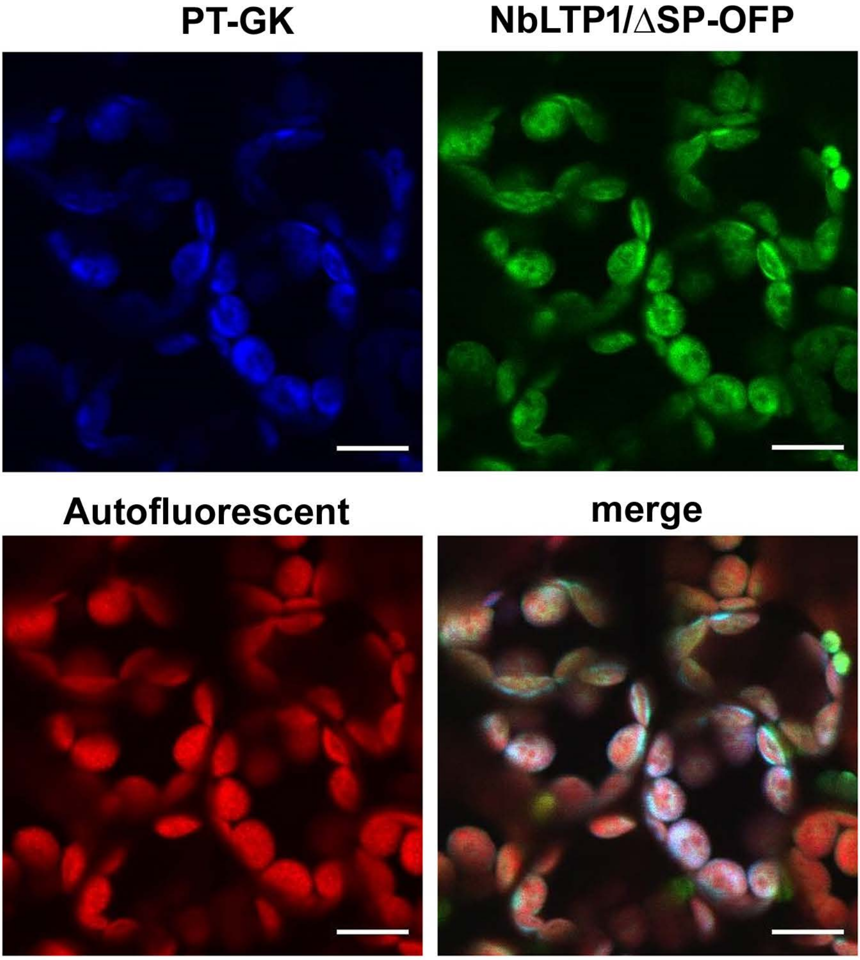

Figure S3: Subcellular localization of NbLTP1/ΔSP-OFP in *N. benthamiana*. OFP-fused NbLTP1/ΔSP mutants were transiently expressed by agro-infiltration in *N. benthamiana* leaves. High resolution images with the chloroplast marker (PT-GK) was obtained under an Olympus Fluoview FV3000 confocal microcope with 543-nm and 633-nm laser excitation. GFP is shown in blue, OFP is shown in green, and the autofluorescent of chloroplasts is in red. Scale bar is 10 μm.

A

N191205_LTP-OFP

SAMPLE: N191205_LTP-OFP
[Thursday, December 5, 2019, 18:57]

Sample Amount: 100.0 picomoles

AAcid #	AAcid ID	R.Time (min)	C.Time (min)	Pmol (raw)	Pmol (-back)	Pmol (+lag)	AAcid ID
1	L	14.90	14.73	4.81	4.81	4.81	Leu
2	T	5.33	5.06	3.90	3.90	3.90	Thr
3	A	0.00	7.14	0.00	0.00	0.00	Ala
4	G	5.57	5.29	1.95	2.71	2.71	Gly

NbLTP1-OFP MAMAGKIACFVVLCMVVAAPHAEA<u>LTCG</u>QVTSKLAP-------

B

12/10/19 17:41 N191205_LTP-OFP

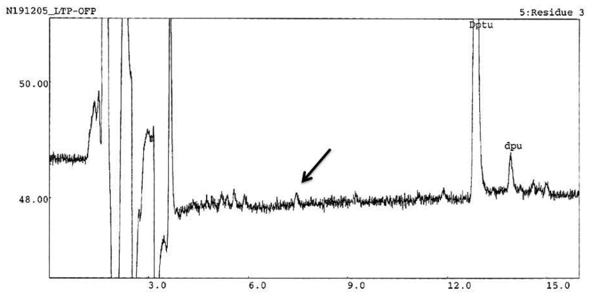

5:Residue 3, Interpolated baseline

Peak No	RT	Peak ID	Type	Height	Pmol Ht
15	12.82	Dptu		124350	90.68
16	13.90	dpu		5048	13.58

C

N191204_OFP

SAMPLE: N191204_OFP
[Wednesday, December 4, 2019, 18:58]

Sample Amount: 100.0 picomoles

AAcid #	AAcid ID	R.Time (min)	C.Time (min)	Pmol (raw)	Pmol (-back)	Pmol (+lag)	AAcid ID
1	V	11.76	11.49	6.22	6.22	6.22	Val
2	S	4.88	4.60	4.18	4.18	4.18	Ser
3	K	14.77	14.44	3.07	3.07	3.07	Lys
4	G	5.57	5.25	2.78	0.00	0.00	Gly

OFP M<u>VSKG</u>EENNMAIIKEFMRFKVRMEG---------

Figure S4: N-terminus sequence determination of the secreted polypeptides. Edman degradation assay was used to determine the N-terminal sequence of the two polypeptides (Figure 4A) isolated from the transiently expressed NbLTP1-OFP on *N. benthamiana* leaves. The N-terminus sequence of the larger band in (A) with the profile of the third cycle in (B) and the lower band in (C). The matched sequence of NbLTP1 in (A) and OFP in (C) is underlined. The ambiguity of the small peak in the third cycle of Edman degradation is indicated with an arrow in (B).
